# The CASP13-CAPRI targets as case studies to illustrate a novel scoring pipeline integrating CONSRANK with clustering and interface analyses

**DOI:** 10.1186/s12859-020-03600-8

**Published:** 2020-09-16

**Authors:** Didier Barradas-Bautista, Zhen Cao, Luigi Cavallo, Romina Oliva

**Affiliations:** 1 KAUST Catalysis Center, King Abdullah University of Science and Technology, Thuwal, Saudi Arabia; 2 Department of Sciences and Technologies, University of Naples “Parthenope”, Centro Direzionale - Isola C4, 80143 Naples, Italy

**Keywords:** Ranking, Docking models, Docking decoys, Prediction, Interface analysis, Consensus, Performance, Clust-CONSRANK, COCOMAPS

## Abstract

**Background:**

Properly scoring protein-protein docking models to single out the correct ones is an open challenge, also object of assessment in CAPRI (Critical Assessment of PRedicted Interactions), a community-wide blind docking experiment. We introduced in the field CONSRANK (CONSensus RANKing), the first pure consensus method. Also available as a web server, CONSRANK ranks docking models in an ensemble based on their ability to match the most frequent inter-residue contacts in it. We have been blindly testing CONSRANK in all the latest CAPRI rounds, where we showed it to perform competitively with the state-of-the-art energy and knowledge-based scoring functions. More recently, we developed Clust-CONSRANK, an algorithm introducing a contact-based clustering of the models as a preliminary step of the CONSRANK scoring process.

In the latest CASP13-CAPRI joint experiment, we participated as scorers with a novel pipeline, combining both our scoring tools, CONSRANK and Clust-CONSRANK, with our interface analysis tool COCOMAPS. Selection of the 10 models for submission was guided by the strength of the emerging consensus, and their final ranking was assisted by results of the interface analysis.

**Results:**

As a result of the above approach, we were by far the first scorer in the CASP13-CAPRI top-1 ranking, having high/medium quality models ranked at the top-1 position for the majority of targets (11 out of the total 19). We were also the first scorer in the top-10 ranking, on a par with another group, and the second scorer in the top-5 ranking. Further, we topped the ranking relative to the prediction of binding interfaces, among all the scorers and predictors. Using the CASP13-CAPRI targets as case studies, we illustrate here in detail the approach we adopted.

**Conclusions:**

Introducing some flexibility in the final model selection and ranking, as well as differentiating the adopted scoring approach depending on the targets were the key assets for our highly successful performance, as compared to previous CAPRI rounds. The approach we propose is entirely based on methods made available to the community and could thus be reproduced by any user.

## Background

Biomolecular complexes, especially protein-protein complexes, underpin almost all biological processes in the cell. The biological function of proteins is in fact defined by their interactions [1, 2]. Inappropriate or altered interactions can lead to disease [2]. Consequently, protein-protein complexes are receiving increasing attention as targets for rational drug design [3–6].

For these and other applications, as well as for understanding the mechanisms underlying biological processes, the relevance of structural information, ideally from high-resolution experimental structures of the complexes of interest, is outstanding. However, experimental structures are available only for a small fraction of known protein-protein complexes, as experimental determination is usually a costly and time-consuming process [7, 8]. Many more protein complex structures could in principle be predicted by computational approaches, in a scenario where protein-protein docking takes on a crucial role complementary to classical structural biology techniques. However, reliably predicting the three-dimensional structure of protein-protein complexes by molecular docking is still an open challenge, with one of the critical steps being the scoring, i.e. the ability to discriminate between correct and incorrect solutions within a wide pool of generated models [9].

Traditionally, scoring functions for protein-protein docking poses calculate a score for each model per se, using energy- and/or knowledge-based algorithms [10–12]. We introduced in the field CONSRANK (CONSensus RANKing), the first pure consensus method [13, 14]. CONSRANK ranks models based on their ability to match the most conserved (or frequent) inter-residue contacts in the ensemble they belong to. We made it available to the scientific community through an advanced interactive web interface at the URL: https://www.molnac.unisa.it/BioTools/consrank/) [15]. The CONSRANK server output includes, besides the models ranking, a list of the inter-residue contacts with relative conservation rates (frequencies in the ensemble). Further, it reports interactive 2D and 3D consensus contact maps (i.e. intermolecular contact maps where the conservation of the contacts in the ensemble is reported on a gray scale [16–19])*.* Once the general output has been generated, the user can choose to perform additional analyses on single models. For instance, CONSRANK can be asked to calculate the inter-molecular contact map for a given model and color consequently the corresponding contacts in the interactive consensus maps. Results relative to the most recently analyzed models are contemporarily reported in the maps in different colors, helping the user to visualize at a glance how much them resemble each other and how well each of them reflects the overall consensus. Later on, we developed Clust-CONSRANK, an algorithm introducing a contact-based clustering of the models as a preliminary step of the CONSRANK scoring process [20]. We conceived of it for the most challenging scoring cases, to the aim of differentiating their top 10 ranked models and possibly enrich them in correct solutions.

Our scoring algorithms could be independently tested and compared to other scoring methods in the Critical Assessment of PRedicted Interactions (CAPRI) experiment. CAPRI organizes blind docking challenges since 2002 [21, 22] and includes a scoring session since 2006 [23]. Modelled after the Critical Assessment of protein Structure Prediction (CASP) [24, 25], it asks its participants to predict the 3D structure of a biological complex before its publication. In the scoring experiment, participants (scorer groups) are invited to download an ensemble of anonymized predicted complexes generated during the docking experiment (100 models for each target are submitted by each predictor to be used for the scoring challenge). Then, they are invited to evaluate the ensemble of models using the scoring function of their choice, and to submit their own 10 top-ranking ones. Therefore, the assessment of scoring functions is made irrespective of the used docking protocols. The time given to the scorers for each target is typically 4–5 days.

In the last two decades CAPRI has been bolstering advancements in the docking field and monitoring its state of the art. One important insight achieved in the latest CAPRI experiments was that a template-based (or comparative) docking procedure, i.e. the prediction of a complex structure based on the structure of a homologous complex, tends to be more successful than an ab initio (or free) docking procedure [26]. On such bases, classification of the targets in easy or difficult (traditionally based on the extent of conformational change upon binding) is now made by the CAPRI assessors mainly based on the availability of good structural templates for a comparative docking procedure [26–28].

At the end of each CAPRI round, the submitted models are assessed by comparing them against the structure of the complex determined by experimental methods (which may be made available to the CAPRI assessors prior to public release through the Protein Data Bank, PDB [29]). Based on root-mean-square deviation (RMSD) values and the fraction of correctly predicted inter-molecular contacts, models are classified by the CAPRI assessors as incorrect or correct. For the correct models a further classification is made, in order of increasing quality it is: acceptable, medium or high quality models [23]. Especially scorers in CAPRI are requested to be able and pick up the high/medium quality models, if available, from the ensembles of models.

We have been participating in all the latest CAPRI scoring experiments, starting from CAPRI30 (joint experiment with CASP11, held during summer 2014 [30]). In the CAPRI rounds 30 to 45, we used CONSRANK in a server-like approach, performing competitively with the other scoring algorithms [26, 28, 30]. However, for the 19 targets of the latest CASP13-CAPRI scoring experiment (CAPRI round 46 joint with CASP round 13, held during summer 2018), we achieved particularly satisfying results. We were indeed first as scorers in the top-1 and top-10 rankings, as well as in the prediction of binding interfaces [27].

Our results in CASP13-CAPRI were the consequence of a novel pipeline that we applied there for the first time. In it, we integrated our scoring tools CONSRANK and Clust-CONSRANK with our analysis tool COCOMAPS [31], all of them being publicly available. In the following, we illustrate such a pipeline, taking advantage of scoring examples from the CASP13-CAPRI experiment, the final aim being that of helping users to apply it in real life scoring cases.

## Results and discussion

### The targets

We submitted scoring predictions for all the 19 targets assessed in the CASP13-CAPRI scoring experiment [27]. Among them, 13 targets were classified as homo-oligomers: 10 homo-dimers, 1 homo-trimer, 1 homo-tetramer and 1 homo-octamer. The remaining 6 targets were hetero-oligomers: 4 hetero-dimers, 1 hetero-teramer and 1 18-mer hetero-complex of the A6,B6,C6 type. They were offered to the CAPRI challenge by individual structural biology laboratories and were high-resolution X-ray structures, with the exception of targets T144, T147 and the T159, being determined by cryo-EM. They covered a spectrum of functions, including enzymes, transporters and channels. Their size was also highly variable, with length of individual subunit ranging from the 72 residues of T142 to the 1589 residues of T151, comprising 4 structural domains. For this last target, models to be scored came from experimental data-free predictions, but also from predictions guided by experimental data, specifically SAXS (small angle x-ray scattering) and XLMS (cross linking mass spectrometry) data, added progressively. This explains the much larger number of models available for this target (4355 vs. an average of 1948 models).

For some targets (T138, T139, T146, T147, T151 and T159), all the distinct interfaces formed with neighboring subunits in the crystal were considered in the assessment. This is the consequence of them being higher order oligomers featuring more than one distinct interface or dimer targets with an ambiguous biological unit assignment. The number of interfaces assessed per target is reported in Table 1. The overall quality score for targets featuring more than one interface was taken as that of the best predicted individual interface for the assembly [27].

**Table 1:**
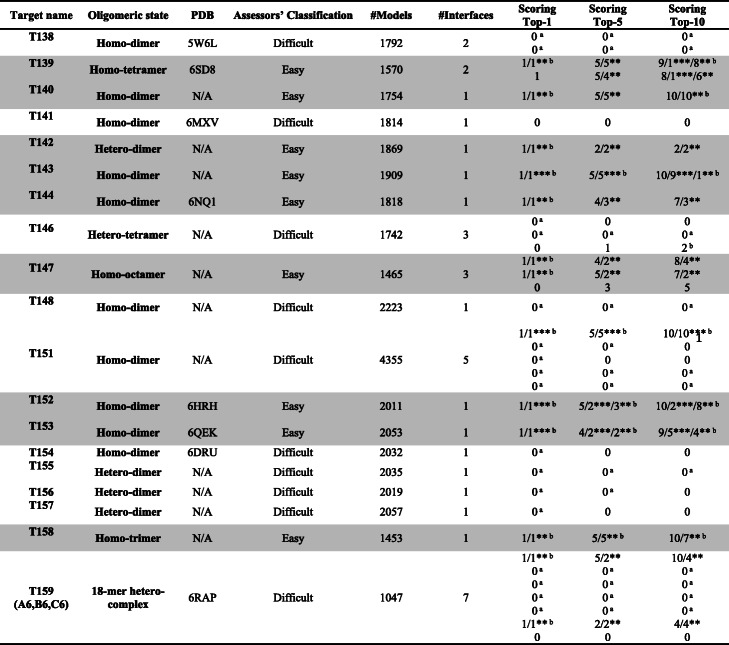
Details on the targets of the CASP13-CAPRI experiment and, in the last three columns, our detailed results as scorers for each target and interface. In the scoring results, it is reported the total number of our correct models (out of the 10 submitted) per target/interface in the top-1, top-5 and top-10 positions; numbers followed by two (**) and three (***) stars represent the subsets of correct models that are of medium and high quality, respectively. Easy targets, according to the assessors’ classification, are shadowed in gray

^a^ No correct solution was submitted by any Scorer

^b^ Best result overall among the Scorers, achieved by our group alone or on a par with others

During the CAPRI assessment, a classification of the targets in easy or difficult to model was made, based on the availability of good structural templates for the full assembly or the main interfaces. In particular, for the scoring, 9 targets (T139, T140, T142, T143, T144, T147, T152, T153 and T158) were classified as easy, while 10 targets (T138, T141, T146, T148, T151, T154, T155, T156, T157 and T159) were classified as difficult. For scorers, the difference between easy and difficult targets is mainly in the level of enrichment in correct models of the combined anonymized ensembles to be evaluated. Availability of good structural templates implies indeed that more predictors will have been able to provide correct models.

This assessors’ classification was largely coincident with the one we made ourselves in the preliminary stage of our assessment, by checking the availability of structural templates by PSIBLAST [32] and HHPRED [33].

### Our performance in the CASP13-CAPRI scoring experiment

Based on the comparison between predictions and the final experimental structures of the target complexes, CAPRI assessors classify correct models, in order of increasing quality, as acceptable, medium or high quality, where the high quality models reproduce at least half of the contacts in the target and have a backbone RMSD within 1.0 Å from it (calculated either on the complex interface or on the smaller size protein after best superimposition of the larger size one). For details see Table S1 and [30]. Assessment for the top-1, top-5, top-10 models submitted by each scorer group per target was provided, with the top-5 ranking being the main one in the CASP-CAPRI experiments.

**Fig. 1:**
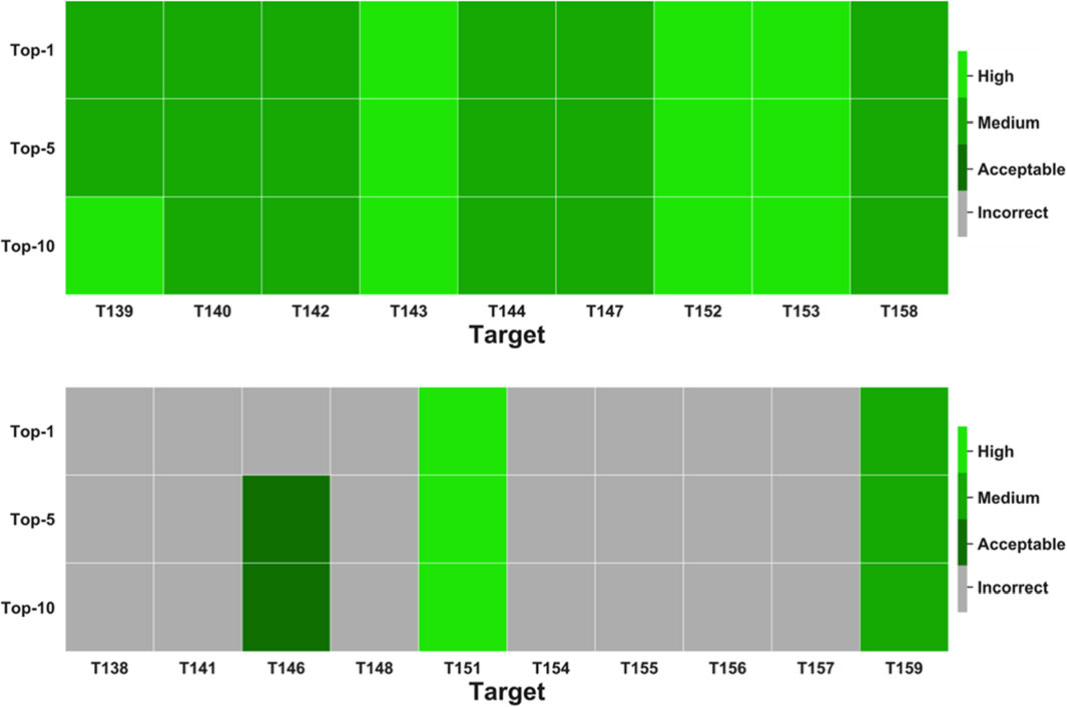
Heatmap reporting our performance as scorers for the 9 easy (top) and 10 difficult (bottom) targets of the CASP13/CAPRI46 joint experiment. The presence of high, medium quality, acceptable or incorrect models in each ranking is reported in a green-to-gray color code

Comparative results of the CASP13-CAPRI are reported in [27]. We were by far the first scorer in the top-1 ranking, having high/medium quality models ranked at the top-1 position for the majority of targets (11 out of the total 19, see Fig. 1 and Table 1), which compares with the 9 high/medium quality, plus 1 acceptable model, of the second scorers in this ranking [27]. Such an achievement is particularly relevant, as this was the first CASP-CAPRI round where scorers were asked to accurately rank their 10 selected solutions from the top to the bottom position, based on the expected quality.

We were also the best performing scorer group in the top-10 ranking, on a par with the Fernandez-Recio’s group, with 11 targets having high/medium quality models, plus 1 target having acceptable models. In the top-5 ranking, we were second after Fernandez-Recio having the same number of targets with correct solutions, 12, but 1 target less with high quality models, 4 vs. 5.

Overall, we were among the top three scorer groups (Fernandez-Recio, Oliva, Zou) submitting correct models within the top-5 models for 12 targets. It is noteworthy that, with the other two top groups, we substantially covered the spectrum of possible methodologies currently used for scoring docking models. Fernandez-Recio’ group uses indeed a physics-based approach [34], while the Zou’s group uses a knowledge-based one [35].

CAPRI assessors also evaluated results of scorer groups separately for the easy and the difficult targets, to investigate whether their comparative performance diverged significantly for the two sets of targets.

Starting with the 9 easy targets, we could rank a high/medium quality model at the top-1 position for all of them. This made us topping again this (top-1) ranking, with the following scorers in the ranking having correct solutions for 8 targets, only 7 of them being of high or medium quality. Again, we were first on a par with Fernendez-Recio in the top-10 ranking, with correct solutions for the 9 targets, 4 of them being of high and 5 of medium quality. And again we were second after Fernendez-Recio in the top-5 ranking having one high/medium quality model less (3 vs. 4).

Focusing on the 10 difficult targets, we were again first, on a par with other 4 scorer groups, in the top-1 ranking, having correct solutions for 2 targets, 1 of high and 1 of medium quality. In the top-5 ranking, we were second, on a par with other 7 groups, and after MDockPP, with correct solutions for 3 targets, 1 of them being of high and 1 of medium quality. Finally, in the top-10 ranking, we had correct solutions for 3 targets, including 1 high and 1 medium quality model. This ranked us 5th on a par with other 9 scorer groups, after 4 groups having all 1 target more (4 overall) with acceptable solutions. Ranking for the difficult targets was indeed quite flattened since the best performing scorer groups successfully predicted the same limited subset of difficult targets [27].

### Our performance in CASP13-CAPRI prediction of binding interfaces

Submitted models were also evaluated by the CAPRI assessors in terms of “prediction of binding interfaces” - an independent category accounting for the correspondence between residues in the predicted interfaces and those observed in the corresponding experimental structures. Interface predictions were evaluated for all the binary associations modes (interfaces) of each target in the top 5 models submitted by both the CAPRI predictors and scorers. To quantify the correctness of residues defining the interfaces of the individual protein components of each binary association mode in the predicted models (i.e. their correspondence to those in the target structure), recall and precision measures were used. For each model, recall (or sensitivity) represented here the fraction of interface residues in the target that was reproduced in the model, while precision (or positive predictive value) represented the fraction of residues at the interface of the model that corresponds to target interface residues.

Group performance in this category was ranked on the basis of the fraction of correctly predicted interfaces (interfaces with both recall and precision ≥0.5), in the top 5 submitted models for each target. Overall, scorers performed better than predictors in this category [27]. In the final ranking, including both CAPRI predictors and scorers, we were in fact the top group, with 47.3% of the interfaces correctly predicted. The second group in this ranking, i.e. Venclovas as a predictor, achieved a 44.6% of correctly predicted interfaces. Overall, only 5 groups (ours, plus Venclovas, Eisenstein, Huang and Zou) achieved correct predictions for at least 40% of the interfaces. Our average recall and precision values for interfaces of individual models were as high as 48.2 and 55.1%, respectively.

### Our novel scoring pipeline

We have been participating in all the latest CAPRI scoring experiments, starting from CAPRI30 (joint experiment with the Critical Assessment of Structure Prediction, CASP11, held during summer 2014 [30]). In all those experiments, we used a server-like approach, which means we ran CONSRANK on the ensemble of models and selected for submission the 10 models top ranked by the algorithm/server, in the exact order they were ranked. No manual intervention was used, apart from checking that selected models did not present too many clashes.

The fact that, based on this simple and automatic approach, we could perform competitively with the other scoring algorithms [26, 28, 30] proves the great potential of CONSRANK itself. However, two clear limitations of the approach emerged from those rounds. The first drawback was the limited diversity of the selected models, which is actually an intrinsic feature of a pure consensus method. The consequence of it was that either our predictions for a given target were all correct, typically for the easy targets, or they were all incorrect, for the difficult targets. The second drawback of our previous approach was the inaccuracy in distinguishing models which were just a reasonable approximation of the real structure (classified as acceptable in CAPRI) from the high/medium quality ones. This is also a critical point, as especially scoring algorithms are requested, in CAPRI and in general, to be able to single out the high quality models, if available. In CAPRI, scorers are indeed ranked primarily based on the number of targets for which they submitted high/medium quality models.

**Fig. 2:**
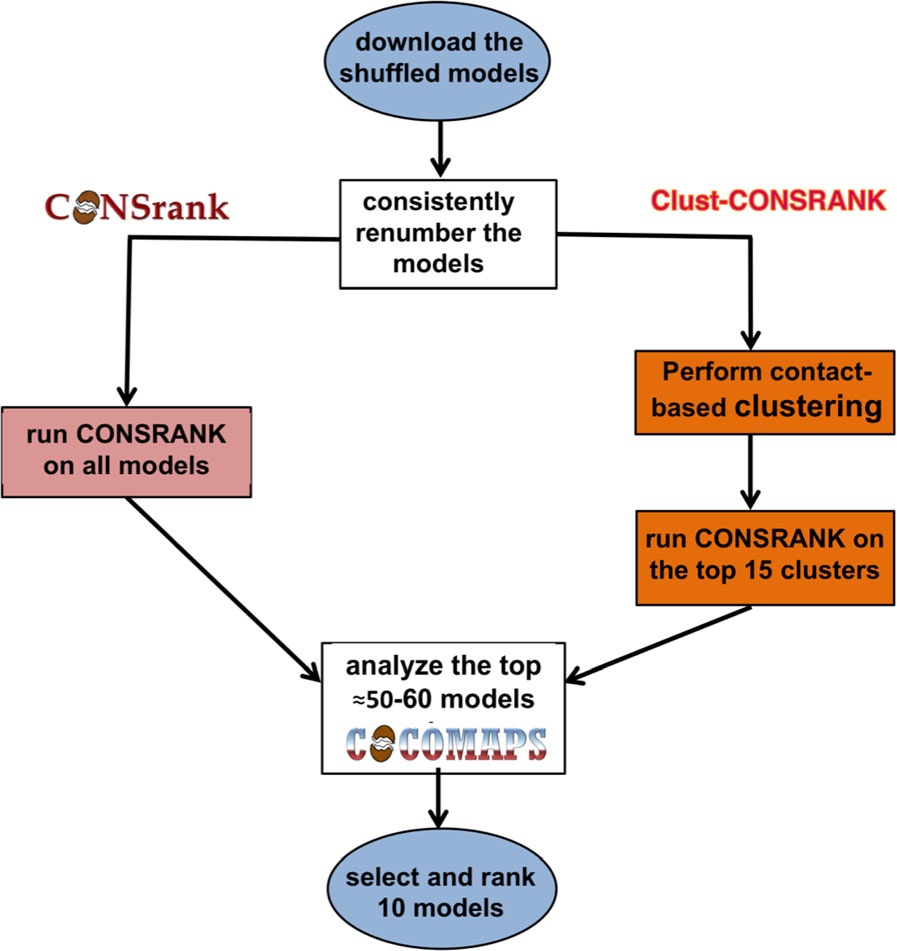
Schematic representation of the novel scoring pipeline used in CASP13-CAPRI, Integrating CONSRANK with Clust-CONSRANK and interface analyses performed by COCOMAPS

In order to overcome the two above limitations of our previous scoring approach, in the latest CASP13-CAPRI experiment we adopted a new pipeline, which led indeed to the very satisfactory results described above. In this new pipeline, we integrated CONSRANK and Clust-CONSRANK (a later implementation of CONSRANK introducing a contact-based clustering of the models as a preliminary step of the scoring process), with the COCOMAPS analysis tool [31] (Fig. 2).

In detail, after the renumbering step, models from the scoring ensembles of each target were subjected to both the CONSRANK and Clust-CONSRANK scoring processes. A total of 50–60 models per target, the top 20–30 models from the CONSRANK ranking and the top 2–3 models ranked by Clust-CONSRANK for each of the 15 most populated clusters, were selected and further analysed in their interface features. To this aim, we used COCOMAPS, a web tool we had specifically developed for the extensive analysis of the interface in experimental and modelled structure of biomolecular complexes [31]. Please notice that this is a quite straightforward step, as a link to COCOMAPS is given in the CONSRANK server output for all the ranked models. A particular attention was given to the extension of the interface, both in terms of interface area (from the buried solvent accessible surface upon complex formation) and number of intermolecular contacts. Models showing a large number of clashes were removed at this stage. Selection of the 10 models for submission from the CONSRANK or Clust-CONSRANK outputs was guided by the sharpness of the consensus emerging from the decoys ensemble, reflected both visually in the consensus map (see the examples below) and quantitatively in the maximum CONSRANK score (the score obtained for the top ranked model). The weaker was the overall consensus, the higher the number of models selected from the Clust-CONSRANK output. This allowed us to diversify our submitted solutions, especially for the difficult targets, thus addressing the first limitation of our previous approach. Ranking of the selected models from the top-1 to the top-10 position was then based on results of the interface analysis. Models exhibiting a more extended contact network and interface area were top ranked. This final step was aimed to address the second limitation of our previous approach, i.e. to try and top rank high quality models instead of just acceptable ones.

Differentiating our approach to add flexibility to the application of CONSRANK alone allowed us to achieve results on a par with the other best performing scorers in CASP13-CAPRI also for the difficult targets. Along the same lines, we applied a basic MD analysis to T159, a 18-mer target for which the renumbering step was unfeasible and consequently CONSRANK could not be ran. In particular, a short in vacuo MD simulation was performed on each of the T159 models, with the aim to test their stability under such conditions. Although successful, we prefer not to speculate much on the approach we used for T159, as it was the first time we tested it and only on 1 target. We need to test it more extensively before possibly deriving general conclusions.

Clearly, results we achieved for the difficult targets in CASP13-CAPRI could not have been accomplished by a server-like use of CONSRANK. In addition, our interface analysis-based method for the final ranking of the 10 selected models, allowed us to top rank high/medium quality models (not simply acceptable ones) for all the easy targets plus two difficult targets. Below, details of the above described approach and relative results are reported and illustrated for a couple of specific cases.

### Details on few targets

To better illustrate the scoring approach we adopted in CASP13-CAPRI, also in light of the achieved results, in the following we will discuss in detail few cases. Such cases had to be selected among those targets having the coordinates of the corresponding experimental structure available to the public to date, through the Protein Data Bank [29] (see Table 1). In the following, we will focus particularly on 1 of our successful cases and 1 of our unsuccessful ones based on the approach illustrated in Fig. 2.

#### T152, a successful case

**Fig. 3:**
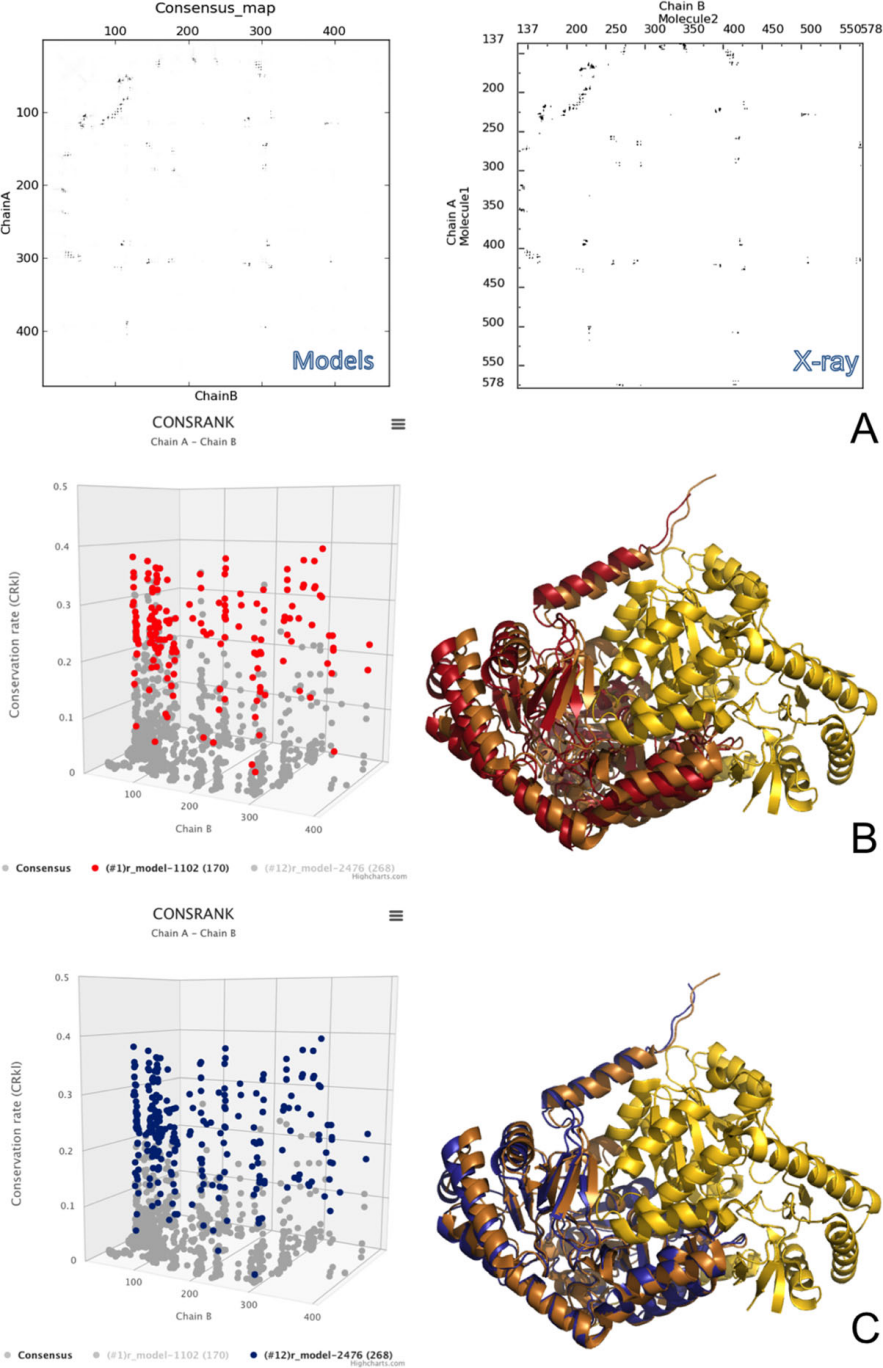
Details on a successful target, T152. **a** Comparison of the consensus map obtained from the scoring models and the contact map generated by COCOMAPS for the corresponding X-ray structure (PDB ID: 6HRH). **b** *Left.* T152 3D consensus map with, in red, the subset of 170 contacts featured by the model ranked 1st by CONSRANK (9th in our submission). *Right.* Comparison of the conformation of the same model (red) with the X-ray structure (gold/copper), after best superimposition of one monomer. **c** *Left.* T152 3D consensus map with, in blue, the subset of 268 contacts featured by the model ranked 11th by CONSRANK (1st in our submission). *Right.* Comparison of the conformation of the same model (blue) with the X-ray structure (gold/copper), after best superimposition of one monomer

T152 was a homodimeric target corresponding to the structure of human erythroid-specific 5′-aminolevulinate synthase, ALAS2 (PDB ID: 6HRH, released on November 2018). T152 was classified as easy in light of the good templates in the same homomeric state available for modelling it. After discarding from the set of 2011 scoring models 90 models having only one chain identifier and 5 models that didn’t match the target sequence, we were left with 1916 models to rank. CONSRANK results evidenced a clear consensus among the analysed models. The value of the CONSANK score for the top ranked model (Maxscore) was indeed as high as 0.243, meaning that the contacts featured by the top-1 ranked model are conserved on average in ≈25% of all the models in the set. In addition, as many as 52 contacts had a conservation rate above 0.3, i.e. were conserved in over 30% of the 1916 models. The clear consensus was also reflected in the corresponding consensus map obtained from the ensemble of models, which showed sharp peaks and negligible background noise (Fig. 3). At the scoring stage, we compared such a consensus map with the contact map of the X-ray structure for ALAS from yeast (PDB ID: 5TXR), a template sharing 43% sequence identity with T152. Not surprisingly, the contacts emerging from the models’ map perfectly matched those of the template, confirming that it (or a similar template) was indeed used by the majority of the predictors. (A posteriori, we could verify that it also matched that of the real structure, see Fig. 3). In light of these results, we decided to select all the 10 models for submission from the CONSRANK output. For the selection, following our novel pipeline, we didn’t just rely on the ranking order of the CONSRANK output. Instead, we analysed the interface of the top 20 CONSRANK models with COCOMAPS. Models ranked 8th, 9th and 15th by CONSRANK were discarded because featuring many (over 25) clashes. The remaining models were ranked based on the number of contacts and on the extension of their interface area, and the top-10 models among them were submitted to CAPRI. For instance, the model ranked 1st by CONSRANK had 170 contacts and an interface area of 3266 Å^2^ and was thus only 9th in our submission, while the model ranked 11th by CONSRANK was the top-1 model in our submission, because featuring 268 contacts and an interface area of 4218 Å^2^ (comparable to the interface area of 4200 Å^2^ in 5TXR). A comparison of the 3D consensus maps (having the conservation rate of each contact on the z-axis) for the above two models is shown in Fig. 3, together with their superimposition on the X-ray structure. In the superimposition, one monomer was optimally superimposed, in order to emphasize the small differences in the conformation/orientation of the other monomer. From the figure it is clear that, although also the model ranked 1st by CONSRANK (in red) was a good approximation of the real structure, the model that we ranked 1st upon interface analysis deviates less from the real structure, especially as concerns the structural motifs (helices in this case) at the interface. The accuracy in the prediction of the interfaces is in fact confirmed by the high performance we achieved in the prediction of the binding interfaces (see above).

Results of the CASP13-CAPRI assessment showed our interface analyses-based approach to be successful. We had a high-quality model as top-1 and two high quality models in the top-5. Only one other scorer group had a high quality model top scored for this target. Our remaining models were of medium quality. The difference between the medium and the high quality models for T152 was clearly small, however it is noteworthy that the interface analyses we performed were able to capture it.

#### T141, an unsuccessful case

T141 was a homodimeric target corresponding to the structure of rhodanese-like family protein from *Francisella tularensis* (PDB ID: 6MXV, released on October 2018). T141 was classified as a difficult target, as it missed good templates both for the complex and for the monomer itself, comprising 2 structurally similar domains, and forming an intertwined homodimer, where domain-domain contacts between monomers are more extensive than those within monomers [27]. Only a few acceptable models were actually submitted overall, by both predictors and servers, for this target.

In our scoring procedure, after discarding 1 model having zero contacts, we were left with 1813 models to rank. The value of the CONSANK score for the top ranked model (Maxscore) was quite low, 0.071, and the top ranked model featured only six inter-residue contacts, meaning that this limited set of six contacts was conserved on average in ≈7% of all the models in the set. Further, the most conserved contact overall had a conservation rate (frequency) in the ensemble of models of only 0.082, meaning that it was conserved in approximately 8% of the 1813 models. These numbers point to the lack of a consensus for the contacts characterizing the interface of this set of models. The appearance of the corresponding consensus map, spread, with a significant background noise, confirmed the absence of a consensus (Figure S2). Therefore, for this target, we differentiated the submission, by selecting 1 model from the CONSRANK output (that we ranked 1st) and 9 models from the Clust-CONSRANK 15 top populated clusters (specifically clusters 1–8 and 11, ranked from the 2nd to the 10th position on the basis of interface features). Unfortunately, the final assessment showed that no one of our models was correct. We show in Figure S2 a comparison between the overall consensus map for the T141 scoring models and those obtained from clusters 1–3 with the contact map of the corresponding experimental structure. From such a comparison, it is clear that there is no correspondence between them. As we could verify a posteriori, the couple of acceptable models present in the ensemble were in clusters of very low population (below 8) or were even singlets. The problem with this target was in particular in the different conformation adopted by the monomers themselves in the set of models to be scored [27]. In this regard, our choice of differentiating the submitted models by applying Clust-CONSRANK was correct. While the models top ranked by CONSRANK (for instance the model we ranked 1st) did not reproduce correctly the conformation of the monomer (in particular the relative orientation of its two domains), models selected from some of the clusters did (cluster 1, 2, 6 and 7 did, see Figure S2). However, also in case of these models, the intertwined homodimer interface was not identified*,* resulting in incorrect solutions.

Clearly our approach, based on the consensus of intermolecular contacts, was penalized here both by the paucity of correct solutions in the scored set and by the dramatic differences in the conformation of the monomers themselves in different models, thus presenting different interfaces for the interaction. It is important to note here that, although Clust-CONSRANK was of utility in this specific case but didn’t eventually allowed to provide correct solutions, it did instead in other cases, such as T147. For T147 we had indeed up to 8 correct models, depending on the specific interface (see Table 1), while only 2 out of the 10 solutions we submitted came from the CONSRANK output. Unfortunately, we cannot discuss this case in further detail, as the coordinates of its experimental structure have not been released yet.

### Can we anticipate the quality of a scoring process?

**Fig. 4:**
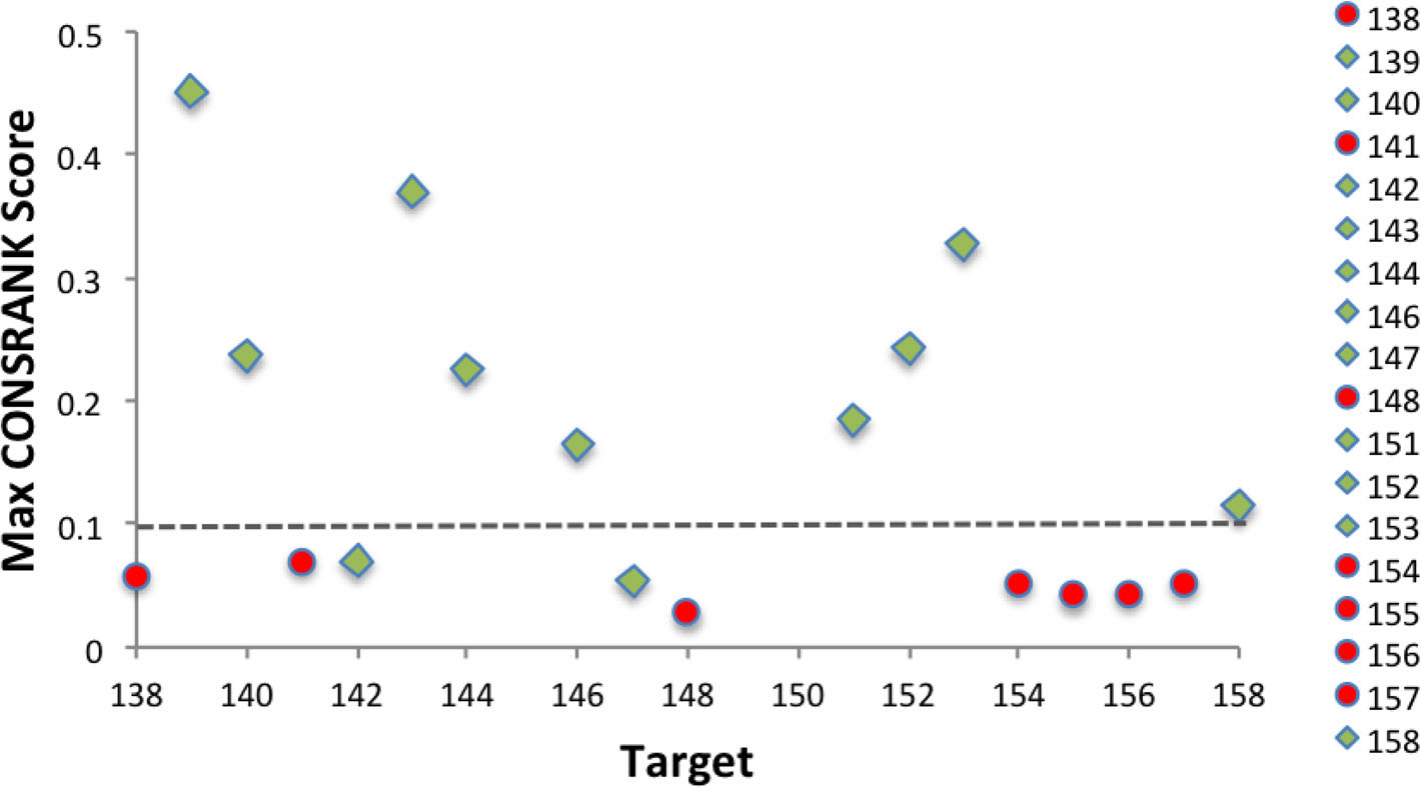
Maximum CONSRANK score (i.e. the score assigned by CONSRANK to the top ranked model) obtained for each of the CASP13-CAPRI scoring targets, but T159. Successful cases for us are shown as green rhombuses, unsuccessful cases as red circles. A dashed line is drawn at the threshold value of 0.1

In a real case scoring scenario, assessment of the models quality based on their comparison with the real complex structure would be obviously unfeasible, as the structure itself would be unavailable. Therefore, the possibility of assessing the scoring quality “a priori” would be of enormous interest. In Fig. 4, we report values of the CONSRANK Maxscore (i.e. the calculated CONSRANK score for the top ranked model) for 18 targets of CASP13-CAPRI (T159 is not included in the plot because, as explained above, it could not be handled with CONSRANK). We showed differently targets that were successful or unsuccessful cases for us. From the figure, a correlation clearly emerges between the CONSRANK Maxscore and the rate of success on the single targets. In particular, all the targets having a Maxscore above ≈0.1 were successful cases for us, with no exception. These include examples of targets classified as difficult, such as T151. For T151, a clear consensus was highlighted by CONSRANK (with a Maxscore of 0.184) pointing to interface 1, for which we submitted indeed 10 high quality models, all selected from the top 16 positions of the CONSRANK output (Figure S4). This was obviously the consequence of having predictions guided by experimental SAXS and XLMS data included in the set of models for T151, as mentioned above.

In all these cases, correct solutions were top ranked by CONSRANK, although in some cases further analyses of their interface were necessary to top rank the high/medium quality models instead of the just acceptable ones. This observation would imply that for a new CAPRI scoring case, for which the reference structure is not yet available, a Maxscore above this threshold would be a guarantee for us of a successful scoring. This observation is corroborated by a similar finding we had for the 22 targets of the CASP11-CAPRI scoring experiment, of which all the 14 successful cases for us featured a Maxscore above ≈0.1 (results shown for the first time in Figure S3).

In addition, in CASP13-CAPRI, two targets, T142 and T147, with Maxscores values below this threshold were also correctly ranked by us. This implies that Maxscore values below 0.1 represent a twilight zone where the scoring can be either correct or, more probably, incorrect. Such targets, T142 and T147, were both classified as easy from the CAPRI assessors [27]. However, for T142, a hetero-dimer, templates were not available for the complex as a whole, which explains the weak consensus obtained from the models submitted for it. The assignment of the oligomeric state for T147 was tentative and corresponded to a high-order oligomer, precisely a homo-octamer adopting a helical assembly of dimeric repeats. Unfortunately for neither of the two targets the corresponding experimental structure is available, therefore we cannot go deeper in the reasons for the success of our scoring approach in these cases. Nevertheless, we can add that our correct solutions for T142 came from the CONSRANK output, confirming that even for such low Maxscore values the consensus among contacts can be, in same cases, significant enough to drive correctly the ranking.

## Conclusions

In the previous CAPRI rounds, we could consistently provide correct models for the easy targets - although not always of high/medium quality -, while we had problems at picking up correct models for the difficult targets [26, 28, 30]. The rate of difficulty of a target is indeed reflected in the level of enrichment (fraction of good solutions) in the models set available for it, to which our scoring algorithm CONSRANK is particularly sensitive. To overcome the above limitation, in the CASP13-CAPRI experiment we introduced a novel scoring pipeline, combining our scoring tools CONSRANK and Clust-CONSRANK with interface analyses performed with our analysis tool COCOMAPS. In one case (T159) even a completely different approach, based on fast MD simulations, was adopted.

As a result of this flexible approach, in the CASP13-CAPRI experiment we had high/medium quality models top ranked for all the 9 targets classified as easy, plus 2 targets classified as difficult (for one more difficult target we could rank acceptable models within the top-5). Differentiating the strategy for the model generation and selection thus allowed us to achieve successful scoring results also for difficult targets, at a rate comparable to the most successful physics-based and knowledge-based algorithms in this round, which are by nature less sensitive to the enrichment of the considered models set. Importantly, additional analyses of the interface allowed us to distinguish models of higher quality from other correct solutions. Such a scoring approach was reported and described here in detail. Since it is based on tools we made available to the community as web servers or standalone programs, it could be applied by any user. It is also noteworthy that a threshold for reliability of the CONSRANK results is consistently emerging (in its Maxscore), defining a “safe zone” where guarantee of a successful scoring process could be given in advance in the context of a real case scoring.

Finally, the quality of the models we submitted in the scoring experiment was also reflected in the high recall and precision achieved in the predicted residues at the interface, making us first also in the ranking for the prediction of binding interfaces, among all predictors and scorers. This last observation opens a possible scenario on the use of CONSRANK for the prediction of the interface residues in a protein complex, in addition to the scoring process itself.

## Methods

### CONSRANK and Clust-CONSRANK

CONSRANK is a consensus algorithm, ranking models based on the conservation of the inter-residues contacts in the ensemble they belong to. Given an ensemble of *n* models of the same biomolecular complex. For each model *i* it first calculates a score as in Eq. 1:

1 $$ {\mathrm{S}}_{\mathrm{i}}=\sum \limits_1^{{\mathrm{M}}_{\mathrm{i}}}{\mathrm{CR}}_{\mathrm{kl}}, $$

where M_i_ is the total number of contacts in model *i* and CR_kl_ is the conservation rate of the contact between residues *k* and *l*, ranging between 0, if the contact is never observed, and 1, if the contact is observed in all the models. Then, it calculates a normalized score, $\overline{S_i}$, as in Eq. 2:

2 $$ {\overline{S}}_i={S}_i/{M}_i. $$

Models are ranked according to their $\overline{S_i}$ value. Two residues are defined in contact if any pair of atoms belonging to the two residues is closer than a cut-off distance of 5 Å. For details, see [13], where test calculations were also performed by varying the cut-off distance in the range 4–10 Å. The software is available as a web application at https://www.molnac.unisa.it/BioTools/consrank/ [15].

Clust-CONSRANK performs a preliminary clustering of the models, to which application of CONSRANK follows for the most populated clusters. For the clustering, a Hamming distance between the models was calculated based on the above defined contacts. The absolute number of different contacts was used as a distance measure between pairs of models. Based on the above calculated metric, a distance n(n-1)/2 sized vector, where n is the number of models, has been obtained by the cluster.pdist function (SciPy library). Elements of this vector represent the Hamming distances between all pairs of models.

At this point, starting from the above distance vector, we generated linkage matrixes by the linkage function (fastcluster library), based on the complete method, having a O(n2) time complexity. The ‘complete’ method assigns:

3 $$ \mathrm{d}\left(\mathrm{u},\mathrm{v}\right)=\max\ \left(\mathrm{dist}\left(\mathrm{u}\left[\mathrm{i}\right],\mathrm{v}\left[\mathrm{j}\right]\right)\right) $$

for all points *i* in cluster *u* and *j* in cluster *v*. It is also known as the Farthest Point Algorithm. Finally, we performed an agglomerative (bottom-up) hierarchical clustering, by the cluster.hierarchy.fcluster function (SciPy library). The Maxclust criterion was used in the clusters generation, i.e. the maximum number of flat clusters, *t*, was set. Maxclust finds automatically a distance value so that no more than t flat clusters are formed. The number of maximum clusters, *t*, was set to 1/10 of the total models available per target/interface, to make the approach independent of the ensemble size. For further details see [20]. Clusters are ranked based on their population and are numbered consecutively, starting from *0*. The top (most populated) 15 clusters were selected here for further analyses.

### Our scoring strategy in CASP13-CAPRI

We submitted scoring predictions for all the 19 targets assessed in the scoring experiment. For each target, we downloaded the shuffled and anonymized scoring set of models, whose number ranged between 1047 (for T159) and 4355 (for T151), and performed a preliminary renumbering step to have them all consistently renumbered, i.e. with corresponding amino acids featuring the same number and chain identifier, which is a fundamental prerequisite for subsequent analyses. At this stage, model files which were empty, featured one only chain identifier or presented sequences not corresponding to those of the target (sequence identity below 90% and coverage below 70%) were discarded. Models presenting zero intermolecular contacts were also discarded. Renumbered models were then scored both with the CONSRANK and Clust-CONSRANK algorithms. The ≈20–30 models with the highest CONSRANK score and the 2–3 top ranked models for the 15 most populated clusters from the Clust-CONSRANK output were checked for clashes (distances within 3 Å) and further analyzed with COCOMAPS, a web tool we had specifically developed for the analysis of the interface in biomolecular complexes [31]. Models for final submission were selected based on the sharpness of the consensus emerging from the decoys ensemble. The weaker the overall consensus, the higher the number of models selected from the Clust-CONSRANK output. Models showing a large number of clashes were removed from the selection. Ranking of the selected models from the top-1 to the top-10 position was based on results of the interface analysis.

For the 18-mer target T159, a completely different approach based on molecular dynamics (MD) was used, as the renumbering step was unfeasible (there was no way to uniquely assign the same identifier to corresponding chains in different models) and consequently CONSRANK could not be ran. For each of the 1047 T159 models, we ran a 100 ps MD simulation in vacuo with GROMACS [36]. MD simulations were performed using the GROMACS 5.0.4 package with the AMBER03 force field. The simulation setting was conceived to minimize the computation time, considering the very large number of systems to be simulated. A box of 60*60*60 Å^3^ was used, including no solvent molecule or counter ion. Simulations were ran in a microcanonical (NVE) ensemble, to avoid perturbations by the thermostat, due to the *vacuo*. Simulations were 0.1 ns (100 ps) long, with a 1 fs time step. The ≈10^2^ models whose simulations didn’t crash were visually inspected and grouped in similar binding arrangements; representative models for the obtained arrangements were selected manually.

## Supplementary information

**Additional file 1 Table S1.** CAPRI criteria for the models assessment, adapted from Lensink et al. (2016) *Proteins*, **84 Suppl 1**, 323–48. f(nat) represents the fraction of contacts in the target (native) that is reproduced in the model, where a contact is defined as any pair of atoms from the ligand (smaller size protein) and the receptor (larger size protein) within 5 Å to each other; L-rms is the root mean square deviation (RMSD) of the backbone atoms of the ligand after optimally superimposition of the receptor in the model and the target structure; I-rms is the RMSD of the backbone atoms of all interface residues after they have been optimally superimposed, where interface residues are those having at least an heavy atom within 10 Å of any atom of the binding partner. **Figure S1.** Comparison of the consensus map obtained from the T152 scoring models and the contact map generated with COCOMAPS for one of the possible templates identified for its modeling (PDB ID: 5TXR), sharing with it a sequence identity of 43% . **Figure S2.** Details on an unsuccessful target, T141. **A**) Comparison of the COCOMAPS contact map for the X-ray structure (PDB ID: 6MXV) with the consensus maps obtained from all the scoring models and the models in the three top populated Clust-CONSRANK clusters. **B**) Superimposition of the conformation of one monomer in the X-ray structure (in gold) and in the model ranked 1st by us, from the CONSRANK output (in cyan) and the model ranked 2nd by us, from the Clust-CONSRANK output (cluster 2, in dark blue). **Figure S3.** Maximum CONSRANK score obtained for the CASP11-CAPRI scoring targets. Successful cases for us are shown as green rhombuses, unsuccessful cases as red circles. **Figure S4.** 2D and 3D CONSRANK contact maps obtained for the T151 models. The Maximum CONSRANK score was as high as 0.184 and several contacts had a conservation rate above 0.3. Contacts featured by the model-ranked 2nd by CONSRANK (original numbering 2126) are shown in red.

## Data Availability

The datasets analysed during the current study are available in the CAPRI homepage repository, at https://www.ebi.ac.uk/msd-srv/capri/round46/, with permission of the CAPRI organizing committee.

## Abbreviations

CAPRI: Critical Assessment of PRedicted Interactions
CASP: Critical assessment of protein structure prediction
MD: Molecular dynamics
PDB: Protein data bank
RMSD: Root-mean-square deviation
