# Supplementary material for: The CASP13-CAPRI targets as case studies to illustrate a novel scoring pipeline integrating CONSRANK with clustering and interface analyses
Source: BMC Bioinformatics. 2020 Sep 16;21(Suppl 8):262. doi: 10.1186/s12859-020-03600-8 (PMC7493188; doi:10.1186/s12859-020-03600-8)
Supplement: Supplementary file 1 — Additional file 1 Table S1. CAPRI criteria for the models assessment, adapted from Lensink et al. (2016) Proteins, 84 Suppl 1, 323–48. f(nat) represents the fraction of contacts in the target (native) that is reproduced in the model, where a contact is defined as any pair of atoms from the ligand (smaller size protein) and the receptor (larger size protein) within 5 Å to each other; L-rms is the root mean square deviation (RMSD) of the backbone atoms of the ligand after optimally superimposition of the receptor in the model and the target structure; I-rms is the RMSD of the backbone atoms of all interface residues after they have been optimally superimposed, where interface residues are those having at least an heavy atom within 10 Å of any atom of the binding partner. Figure S1. Comparison of the consensus map obtained from the T152 scoring models and the contact map generated with COCOMAPS for one of the possible templates identified for its modeling (PDB ID: 5TXR), sharing with it a sequence identity of 43% . Figure S2. Details on an unsuccessful target, T141. A) Comparison of the COCOMAPS contact map for the X-ray structure (PDB ID: 6MXV) with the consensus maps obtained from all the scoring models and the models in the three top populated Clust-CONSRANK clusters. B) Superimposition of the conformation of one monomer in the X-ray structure (in gold) and in the model ranked 1st by us, from the CONSRANK output (in cyan) and the model ranked 2nd by us, from the Clust-CONSRANK output (cluster 2, in dark blue). Figure S3. Maximum CONSRANK score obtained for the CASP11-CAPRI scoring targets. Successful cases for us are shown as green rhombuses, unsuccessful cases as red circles. Figure S4. 2D and 3D CONSRANK contact maps obtained for the T151 models. The Maximum CONSRANK score was as high as 0.184 and several contacts had a conservation rate above 0.3. Contacts featured by the model-ranked 2nd by CONSRANK (original numbering 2126) are shown in red. [file 12859_2020_3600_MOESM1_ESM.docx]

**Table S1.** CAPRI criteria for the models assessment, adapted from Lensink et al. (2016) *Proteins*, **84 Suppl 1**, 323-48. f(nat) represents the fraction of contacts in the target (native) that is reproduced in the model, where a contact is defined as any pair of atoms from the ligand (smaller size protein) and the receptor (larger size protein) within 5 Å to each other; L-rms is the root mean square deviation (RMSD) of the backbone atoms of the ligand after optimally superimposition of the receptor in the model and the target structure; I-rms is the RMSD of the backbone atoms of all interface residues after they have been optimally superimposed, where interface residues are those having at least an heavy atom within 10 Å of any atom of the binding partner.

| **f(nat)** |  | **L-rms (Å)** |  | **I-rms (Å)** | **Assessment** |
| --- | --- | --- | --- | --- | --- |
| ≥ 0.5 | AND | ≤ 1.0 | OR | ≤ 1.0 | High |
| ≥ 0.3 | AND | < 1.0-5.0] | OR | < 1.0-2.0] | Medium |
| ≥ 0.1 | AND | < 5.0-10.0] | OR | < 2.0-4.0] | Acceptable |
| < 0.1 | AND | > 10.0 | OR | > 4.0 | Incorrect |

**
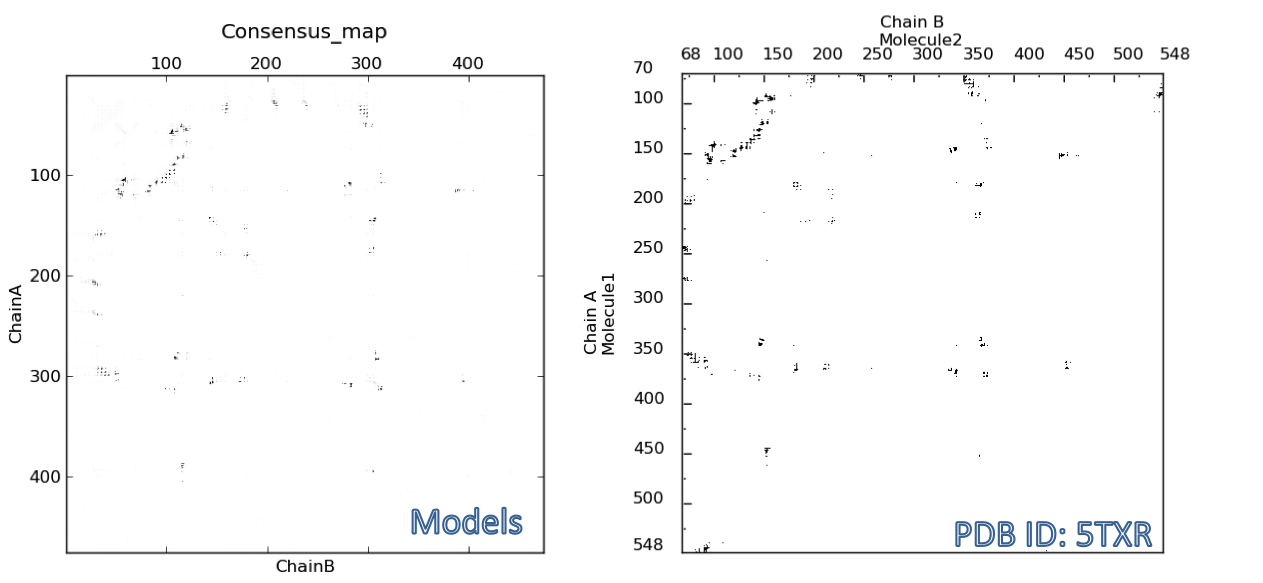
**

**Figure S1.** Comparison of the consensus map obtained from the T152 scoring models and the contact map generated with COCOMAPS for one of the possible templates identified for its modeling (PDB ID: 5TXR), sharing with it a sequence identity of 43% .


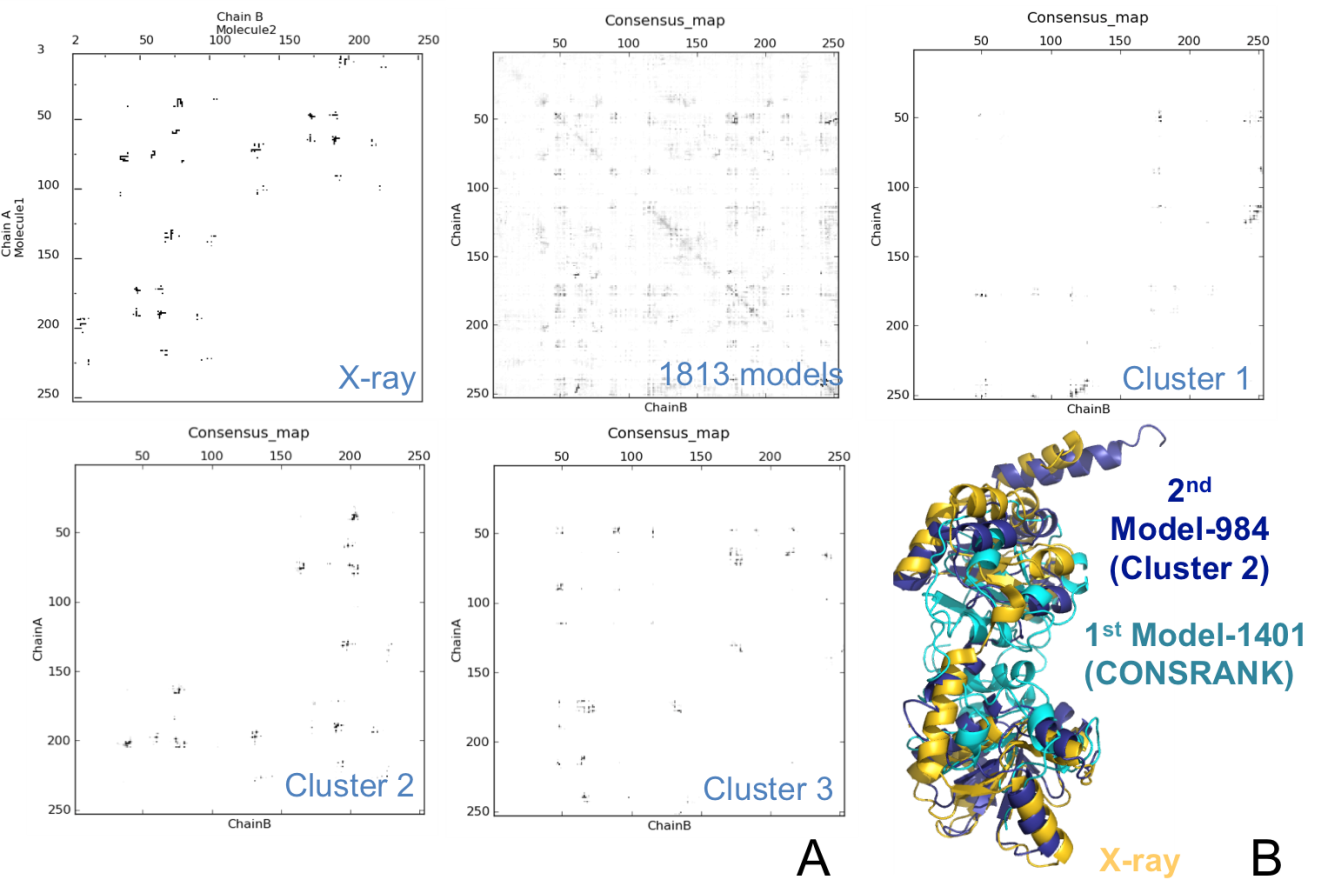


**Figure S2.** Details on an unsuccessful target, T141. **A**) Comparison of the COCOMAPS contact map for the X-ray structure (PDB ID: 6MXV) with the consensus maps obtained from all the scoring models and the models in the three top populated Clust-CONSRANK clusters. **B**) Superimposition of the conformation of one monomer in the X-ray structure (in gold) and in the model ranked 1^st^ by us, from the CONSRANK output (in cyan) and the model ranked 2^nd^ by us, from the Clust-CONSRANK output (cluster 2, in dark blue).

**
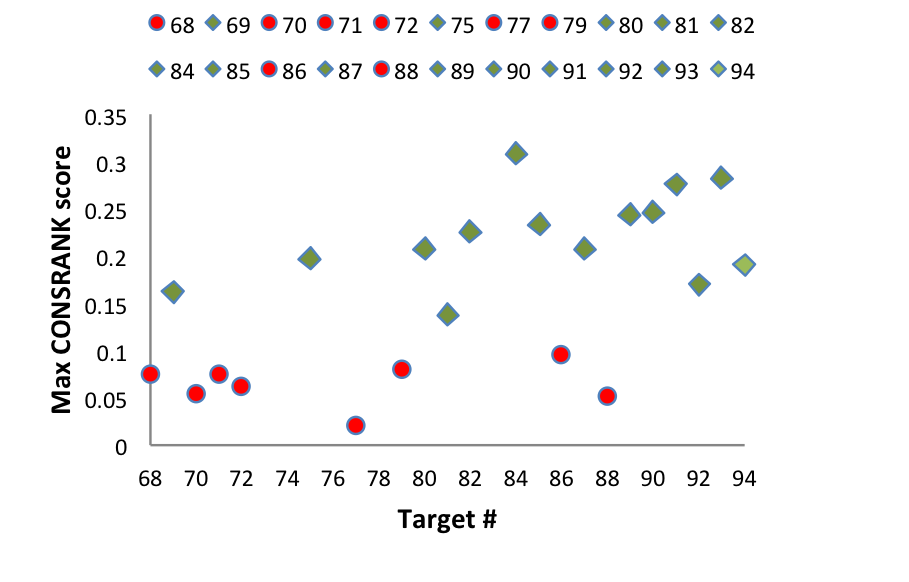
**

**Figure S3.** Maximum CONSRANK score obtained for the CASP11-CAPRI scoring targets. Successful cases for us are shown as green rhombuses, unsuccessful cases as red circles.

**
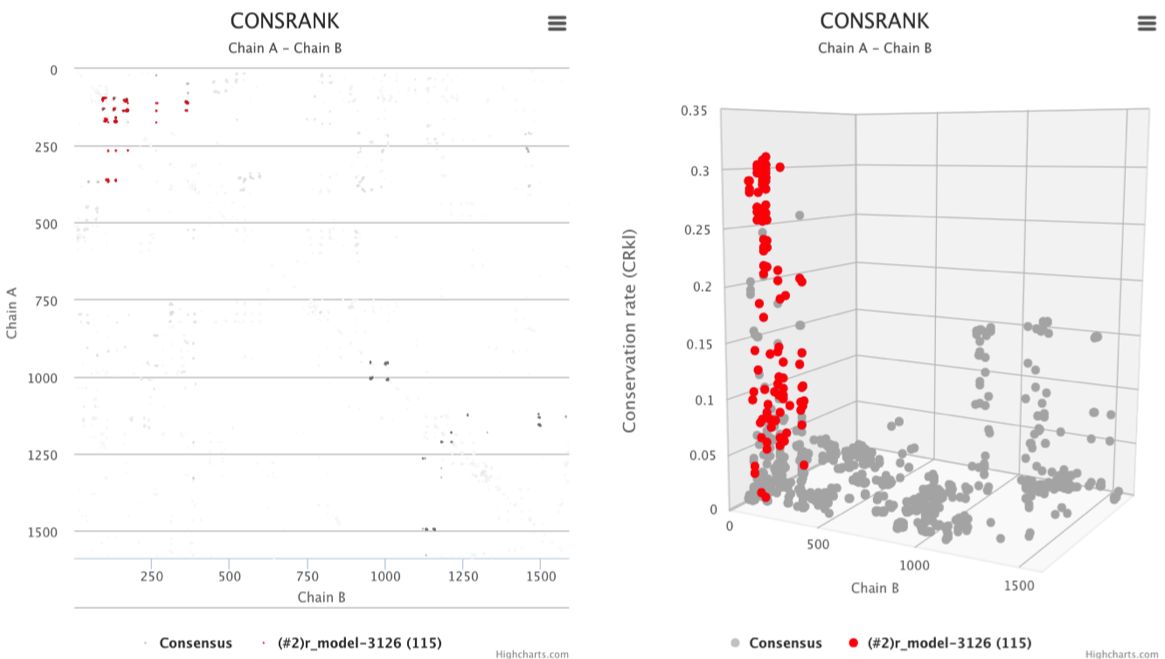
**

**Figure S4.** 2D and 3D CONSRANK contact maps obtained for the T151 models. The Maximum CONSRANK score was as high as 0.184 and several contacts had a conservation rate above 0.3. Contacts featured by the model-ranked 2^nd^ by CONSRANK (original numbering 2126), are shown in red.
